# Effect of astigmatism on visual outcomes after multifocal intraocular lens implantation: a systematic review and meta-analysis

**DOI:** 10.3389/fmed.2023.1214714

**Published:** 2023-11-28

**Authors:** Wenqian Shen, Baoxian Zhuo, Limei Zhang, Jiying Shen, Dongmei Ma, Jin Yang

**Affiliations:** ^1^Department of Ophthalmology and the Eye Institute, Eye and Ear, Nose, and Throat Hospital, Fudan University, Shanghai, China; ^2^Key National Health Committee Laboratory of Myopia, Fudan University, Shanghai, China; ^3^Laboratory of Myopia, Chinese Academy of Medical Sciences, Shanghai, China; ^4^Key Laboratory of Visual Impairment and Restoration, Eye & ENT Hospital, Fudan University, Shanghai, China; ^5^Department of Ophthalmology, Shanghai Heping Eye Hospital, Shanghai, China

**Keywords:** astigmatism, multifocal intraocular lens, cataract surgery, meta-analysis, systematic review

## Abstract

**Purpose:**

To investigate the effects of postoperative astigmatism on the visual outcomes following presbyopia-correcting surgery with multifocal intraocular lens implantation.

**Methods:**

A comprehensive literature search was conducted using PubMed, Embase, and Web of Science for articles published until January 2023. Additionally, we included retrospective case series and prospective comparative studies. The combined mean difference (MD) with 95% confidence intervals (CI) and odds ratio (OR) with 95% CI were used to express continuous and categorical outcomes, respectively. All statistical analyses were performed using Review Manager (version 5.4.1).

**Results:**

We included nine eligible studies that analyzed 3,088 eyes. The proportion of eyes with useful postoperative visual acuity (logMAR ≤ 0.20) and residual astigmatism significantly differed with respect to the magnitude of astigmatism and presence/absence of blurred vision (*p* < 0.001 for both). Additionally, the mean uncorrected distance visual acuity (MD, 0.14; 95% CI, 0.06 to 0.21; *p* = 0.0003) and uncorrected intermediate visual acuity (MD, 0.07; 95% CI, 0.00 to 0.13; *p* = 0.04), but not the uncorrected near visual acuity (MD, 0.02; 95%CI-0.01 to 0.05; *p* = 0.17), significantly differed according to the magnitude of astigmatism.

**Conclusion:**

Astigmatism, even at low levels (≥ 0.5D), has a significant effect on visual outcomes, especially on UDVA and UIVA, following multifocal intraocular lens implantation. Accurate preoperative and postoperative evaluation of astigmatism is important.

## 1 Introduction

Astigmatism is a refractive condition in which parallel rays of light entering the eye do not converge to a single focal point; further, it can be categorized as corneal, lenticular, and retinal astigmatism (1). In China, >47% of patients with cataract have preexisting astigmatism of >1.0 diopters (D) (2, 3); moreover, ≈90% of these patients exhibit astigmatism of ≥0.5 D (4). Therefore, it is important to minimize postoperative residual astigmatism (RA) in these patients to allow excellent visual acuity (VA) and satisfactory vision quality.

Multifocal intraocular lenses (MIOLs) are widely used to treat patients with cataracts and presbyopia. MIOLs can be refractive, diffractive, or a combination of both (5). MIOL implantation is widely considered as among the most effective methods for allowing favorable postoperative vision at all distances; further, it allows generally good satisfaction and spectacle independence. However, neuroadaption, lens dislocation, residual refractive error, and lens opacification may limit the visual performance of MIOL, leading to blurred vision and photic phenomena (6). Specifically, astigmatism is a crucial limiting factor that significantly influences the performance of MIOLs (7–9), while individuals with uncorrected astigmatism or astigmatism with coma appear to be more perceptually adapted to their astigmatism (10–13). However, the mechanisms underlying the vulnerability of eyes with MIOLs to astigmatism remains unclear, with some studies attributing this phenomenon to the intricate light diffraction with MIOLs and eyes (14). Moreover, a previous study using an experimental optical system reported that MIOLs led to interference of the posterior and anterior lines of the nearest and next focuses, respectively, when astigmatism created focal lines for each focal spot in the IOL; furthermore, light energy passed through the expanded conoid of Sturm, which could be attributed to the multiple foci of the MIOLs (15).

The visual outcomes of patients with astigmatism following MIOL implantation and the effect of astigmatism on MIOL performance remain inconclusive. Therefore, this systematic review and meta-analysis aimed to determine the effect of astigmatism on MIOL, which could inform the clinical treatment of patients with presbyopia and cataracts.

## 2 Methods

### 2.1 Study selection

Full texts or abstracts for studies that evaluated the clinical outcomes of MIOL implantation after cataract surgery were eligible for inclusion. The inclusion criteria for studies were as follows (1): population and intervention: patients who underwent cataract surgery followed by MIOL implantation (2); study design: observational studies, prospective or retrospective studies, randomized controlled trials (RCT), controlled studies, or case series; and (3) outcome measurement: visual acuity (logarithm of the minimum angle of resolution [logMAR]) and astigmatism (in diopters).

### 2.2 Method of literature search

A literature search was performed using PubMed, Embase, and Web of Science databases. The search terms were as follows: (“astigmatism”) and (“multifocal intraocular lens” or “multifocal IOL” or “MIOL”). Additionally, we performed a manual search of the reference lists of the included articles and relevant systematic reviews to identify additional studies. The searches were not limited by publication year, study design, or language. The first author independently performed the selection of studies, including searching, duplicate checking, title and abstract screening, and full-text article screening based on the eligibility criteria.

### 2.3 Measurement outcomes

The magnitude of astigmatism and the following visual outcomes were documented: uncorrected distance visual acuity (UDVA), uncorrected intermediate visual acuity (UIVA), uncorrected near visual acuity (UNVA), and RA. We only extracted logMAR visual acuity scores for the meta-analysis. In case the study did not report the mean VA, we used the proportion of eyes with useful VA (logMAR ≤ 0.20, according to the necessary distance VA to drive a car and the necessary near VA to read a newspaper). A low proportion of eyes with useful VA indicates that astigmatism has a significant effect on MIOL performance.

### 2.4 Data extraction

We extracted the following basic information regarding the included studies: first author, publication year, region, study design, number of eyes in different groups, follow-up period, and outcome indicators. If the study reported the outcome indicators at several time points, this study included the data collected at 3 postoperative months or close to each other.

### 2.5 Quality evaluation

The Newcastle–Ottawa scale (NOS) was used to assess the quality of the methodology in six cohort studies. The NOS comprised the following three broad domains: selection, comparability, and outcome. The highest possible total NOS score was 9, with a score of >7 indicating high quality. In addition, the Methodological Index for Non-Randomized Studies was used to assess the methodological quality of the selected non-randomized studies. The Agency for Healthcare Research and Quality (AHRQ) methodology checklist was used for the cross-sectional study, which included 11 items with a summary judgment.

### 2.6 Statistical analysis

Statistical analyses were performed using Review Manager software (RevMan, version 5). Between-study heterogeneity was tested using chi-square statistics, with I^2^ > 50% and *p* < 0.05 indicating statistical significance. Fixed-and random-effects models were used in the absence and presence of heterogeneity, respectively. The odds ratios (ORs) were calculated for the proportion of prediction errors for each method. OR < 1 indicates a lower rate of the method’s outcome. Statistical significance was set at *p* < 0.05.

## 3 Results

### 3.1 Systematic review

**Figure 1:**
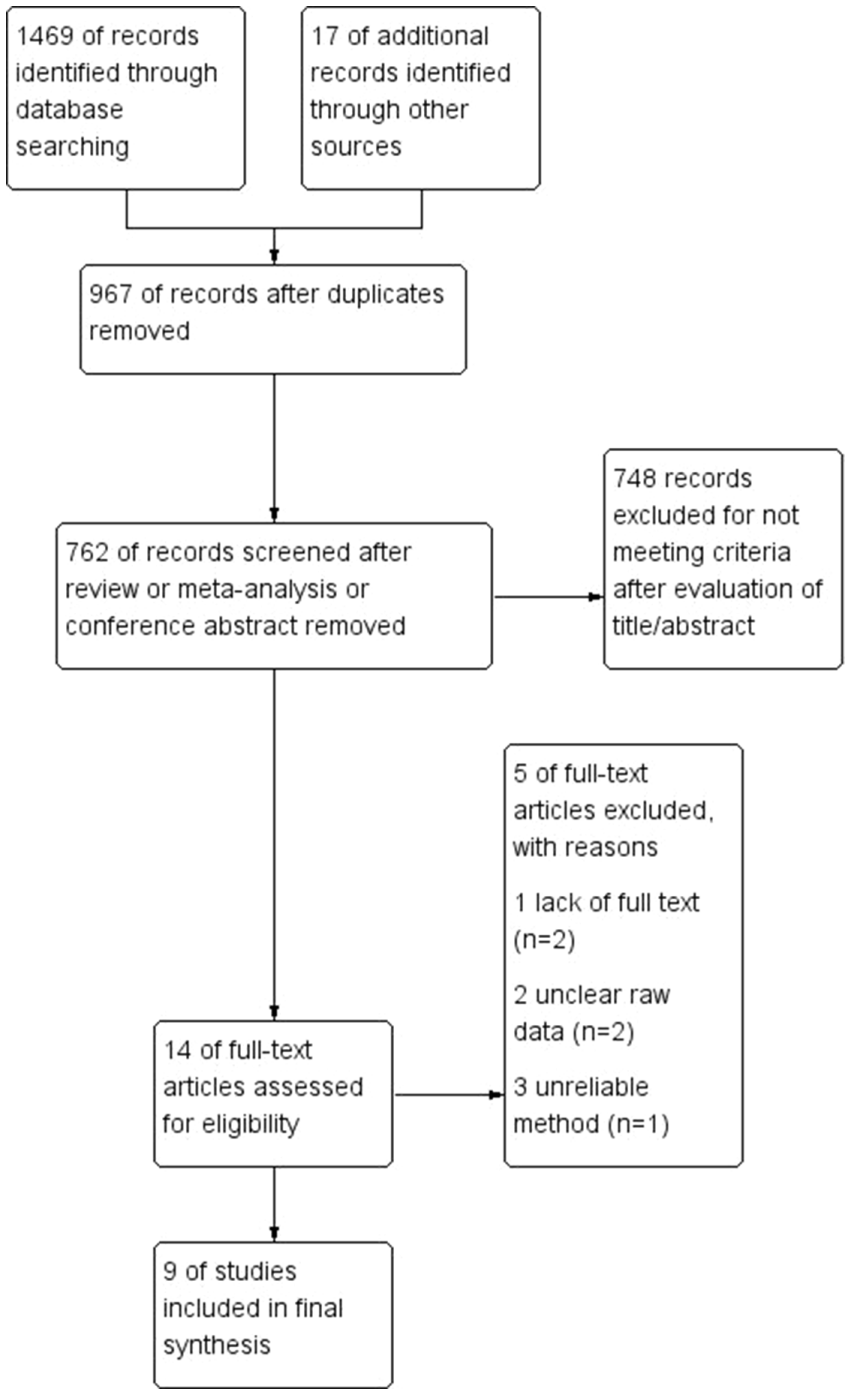
Flow chart depicting the selection of included studies.

The initial search yielded 1,486 articles. After removing duplicate studies, 967 articles remained; among them, 748 articles were excluded after title and abstract screening. There were no relevant meta-analyses. After full-text screening based on the eligibility criteria, we included nine studies that assessed the effects of astigmatism on MIOLs. Figure 1 shows a flow diagram of the selection process.

### 3.2 Study characteristics

Table 1 presents the main characteristics of the nine studies; among them, there were six retrospective case series and three prospective comparative studies. These studies included a total of 3,088 eyes. Three, one, and four studies were conducted in Europe, the United States, and Asia, respectively.

**Table 1:** Characteristics of the included studies.

Study^*^	Country	Year	Eyes, *n*	Study Quality^†^	Design	Range of astigmatism	Model of the MIOL
De Vries	Netherland	2011	76	7	Cohort study	0–2.0D	AcrySof IQ ReSTOR, model SA60D3, SN60D3, SN6AD3, SN6AD1; Tecnis, model ZMA00, ReZoom
Gundersen	USA	2016	416	7	Cohort study	0–1.5D	Rayner Sulcoflex, model 653T
Hao	China	2018	34	7	Cohort study	0–1.0D	AcrySof IQ ReSTOR Toric-2 IOL; AcrySof IQ ReSTOR IOL
Hayashi	Japan	2010	90	-	Non-randomized study	0–2.0D	AcrySof IQ ReSTOR, model SN6AD1, SN6AD3
Hayashi	Japan	2020	150	-	Cross-sectional study	0–1.5D	AcrySof IQPanOptix, model TFNT00; AcrySof IQ ReSTOR, model SA60D1
Hayashi	Japan	2000	60	-	Non-randomized study	0–2.5D	Allergan, model PA154N
Richard	UK	2016	234	8	Cohort study	0–1.5D	Lentis M plus, LS-312 MF30
Steven	UK	2020	1985	7	Cohort study	0–2.0D	Not mentioned
Woodward	USA	2009	43	7	Cohort study	0–2.0D	AcrySof ReSTOR IOL; Tecnis, model ReZoom

* First author.

† Result of the 9-star Newcastle-Ottawa Scale.

### 3.3 Quality assessment

The methodology quality of the six included cohort studies was assessed using the NOS (Table 1). Among the six retrospective studies, one and five studies had an NOS score of 8 and 7, respectively. The MINORS of the included non-randomized studies and the AHRQ methodology checklist of quality assessment of the included cross-sectional study are displayed in Tables 2, 3, respectively.

**Table 2:** Methodological index for non-randomized studies (MINORS).

MINORS score Author	Year	1	2	3	4	5	6	7	8	9	10	11	12	Total
Hayashi et al.	2000	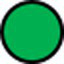	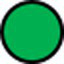	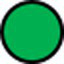	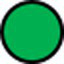	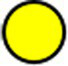	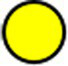	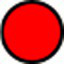	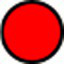	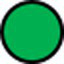	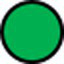	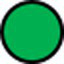	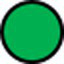	18
Hayashi et al.	2010	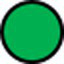	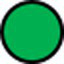	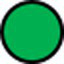	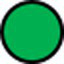	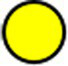	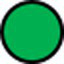	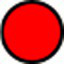	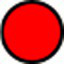	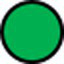	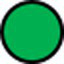	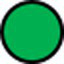	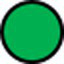	19

Items 1–12 represent: 1, a clearly stated aim; 2, inclusion of consecutive patients; 3, prospective collection of data; 4, endpoints appropriate to the aim of the study; 5, unbiased assessment of the study endpoint; 6, follow-up period appropriate to the aim of the study; and 7, loss to follow-up less than 5%; 8, prospective calculation of the study size. An item scored 0 means not mentioned, 1 means reported but inadequate, and 2 means reported and adequate. The total score was 16 for self-controlled studies. Use red for 0, yellow for 1 yellow, and green for 2.

**Table 3:** Quality assessment of the cross-sectional study with AHRQ methodology checklist.

Item Yes No Unclear	Hayashi 2020
(1) Define the source of information (survey, record review)	1
(2) List inclusion and exclusion criteria for exposed and unexposed subjects (cases and controls) or refer to previous publications	1
(3) Indicate time period used for identifying patients	1
(4) Indicate whether or not subjects were consecutive if not population-based	0
(5) Indicate if evaluators of subjective components of study were masked to other aspects of the status of the participants	1
(6) Describe any assessments undertaken for quality assurance purposes (e.g., test/retest of primary outcome measurements)	1
(7) Explain any patient exclusions from analysis	0
(8) Describe how confounding was assessed and/or controlled.	1
(9) If applicable, explain how missing data were handled in the analysis	0
(10) Summarize patient response rates and completeness of data collection	0
(11) Clarify what follow-up, if any, was expected and the percentage of patients for which incomplete data or follow-up was obtained	1
Score	7
Quality of Study	Moderate

### 3.4 Clinical outcomes

#### 3.4.1 Useful postoperative visual acuity

**Figure 2:**
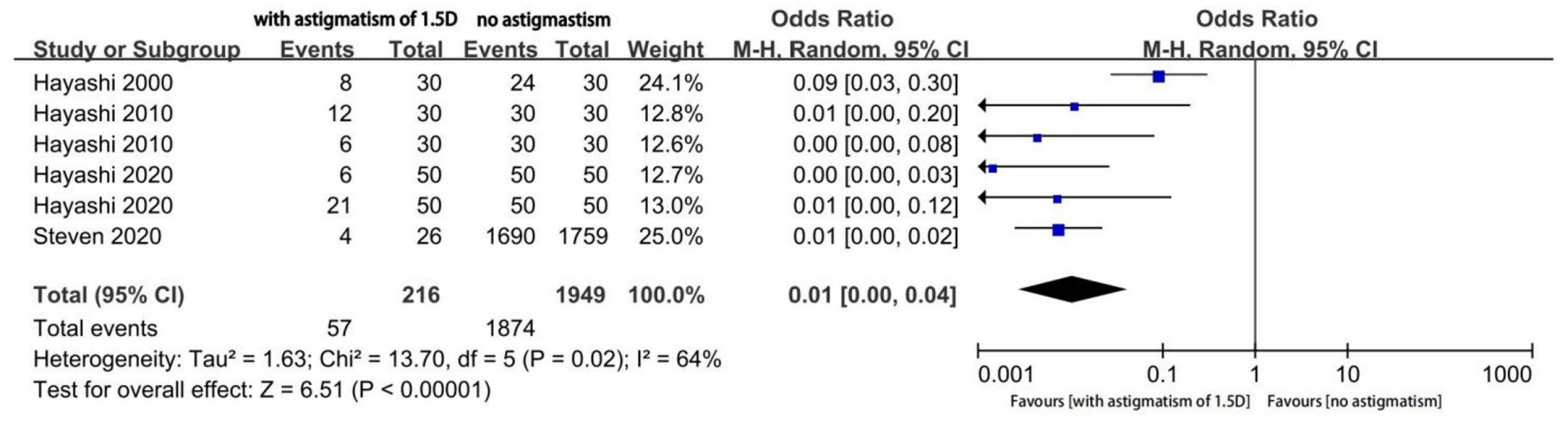
Forest plot of the proportion of eyes with a useful postoperative visual acuity (logMAR ≤0.20) when astigmatism was 1.5D.

**Figure 3:**
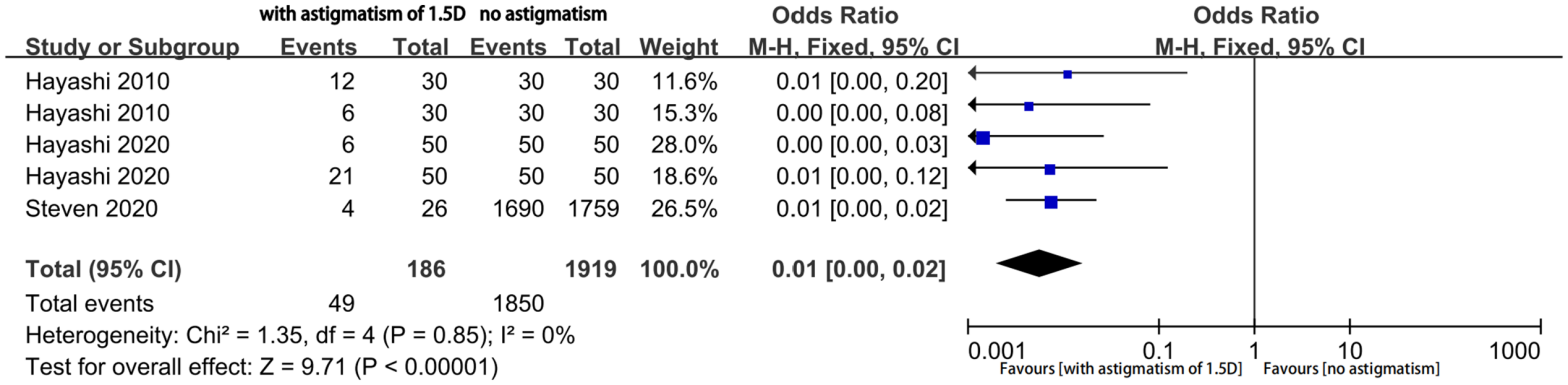
Forest plot of the proportion of eyes with a useful postoperative visual acuity (logMAR ≤0.20) when astigmatism was 1.5D, following removal of the study conducted in 2000 by Hayashi et al.

**Figure 4:**
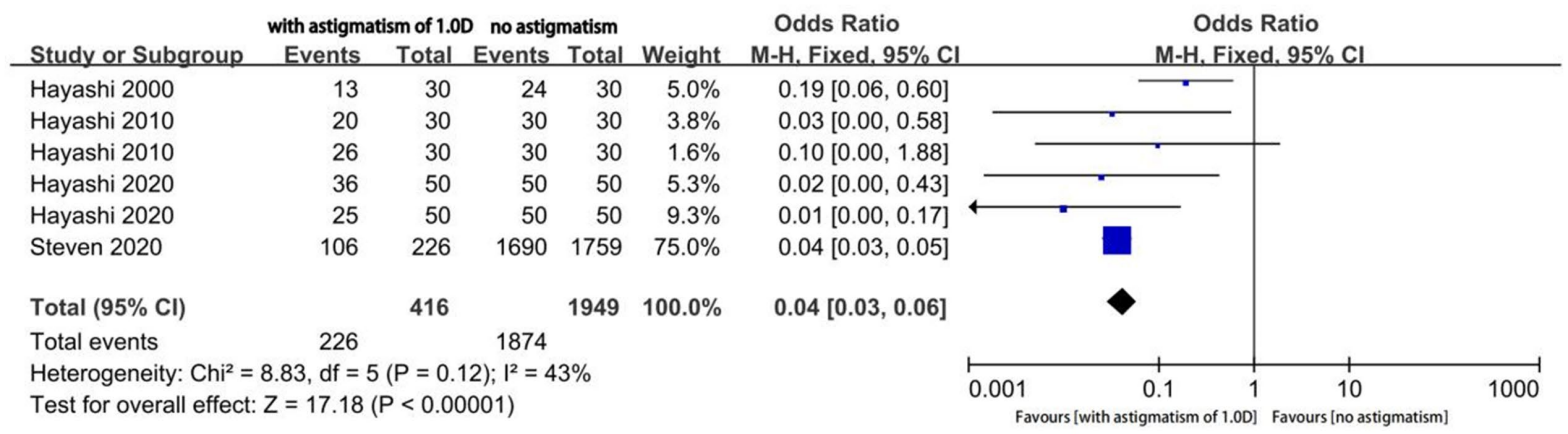
Forest plot of the proportion of the eyes with a useful postoperative visual acuity (logMAR ≤0.20) when astigmatism was 1.0D.

**Figure 5:**
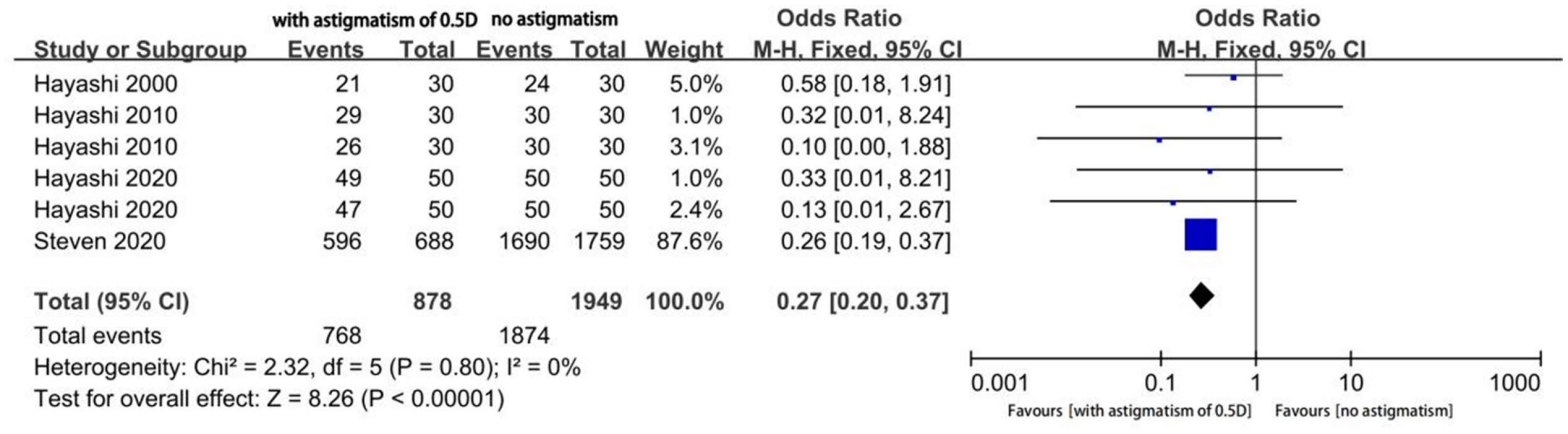
Forest plot of the proportion of the eyes with a useful postoperative visual acuity (logMAR ≤0.20) when astigmatism was 0.5D.

Four studies reported the proportion of eyes with a useful postoperative VA (logMAR ≤ 0.20); among them, two studies reported the outcomes of two different types of MIOLs. We performed an analysis of different magnitudes of astigmatism (1.5D, 1.0D, and 0.5D) to explore the tolerance of eyes with MIOL to astigmatism. The proportion of eyes with a useful VA was significantly different between eyes with astigmatism of 1.5D and eyes with no astigmatism (OR, 0.01; 95% confidence interval [CI], 0.00–0.04; *p* < 0.001; I^2^ = 64%; Figure 2). We further performed sensitivity analysis and observed slight changes in the significance of the difference when we deselected any included studies; this indicated good stability. Moreover, there was a considerable decrease in heterogeneity (OR, 0.01; 95% confidence interval [CI], 0.00–0.02; *p* < 0.001; I^2^ = 0%; Figure 3) when we excluded the study by Hayashi et al. performed in 2000 (16), which may have been the source of the heterogeneity. Significant differences were also found in the proportion of eyes with a useful VA between eyes with astigmatism of 1.0D (OR, 0.04; 95% CI, 0.03–0.06; *p* < 0.001; I^2^ = 43%; Figure 4), and 0.5D (OR, 0.04; 95% CI, 0.03–0.06; *p* < 0.001; Figure 5) and eyes without astigmatism. The I^2^ value was relatively low, which indicated a quantitatively small heterogeneity.

#### 3.4.2 Residual astigmatism

**Figure 6:**
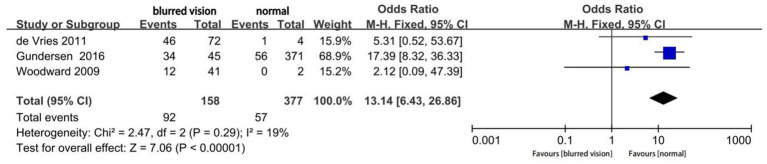
Forest plot of the proportion of residual astigmatism >1.0D.

Three studies reported the proportion of residual astigmatism >0.75D in eyes with blurred vision after MIOL implantation (Figure 6). The proportion of residual astigmatism significantly differed according to the presence or absence of blurred vision (OR, 13.14; 95% CI 6.43–26.86; *p* < 0.0001).

#### 3.4.3 Mean postoperative visual acuity

**Figure 7:**
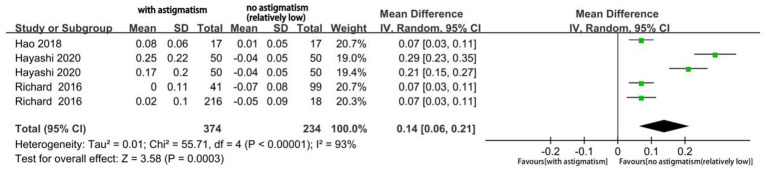
Forest plot of uncorrected distance visual acuity (UDVA).

Three studies reported the mean postoperative UDVA, UIVA, and UNVA. Hayashi et al. reported the outcomes of two different types of multifocal IOLs, while Richard et al. explored the effects using two different classification methods of astigmatism (refractive and corneal astigmatism). Figure 7 shows the mean postoperative UDVA at selected time points. UDVA significantly differed according to the magnitude of astigmatism (mean difference [MD], 0.14; 95% CI, 0.06 to 0.21; *p* = 0.0003). I^2^ was 93%, which indicated a large heterogeneity. Subsequently, we performed a subgroup analysis according to the type of implanted IOL (trifocal IOL and bifocal IOL). However, the source of heterogeneity could not be identified. We also performed sensitivity analysis and observed good stability.

**Figure 8:**
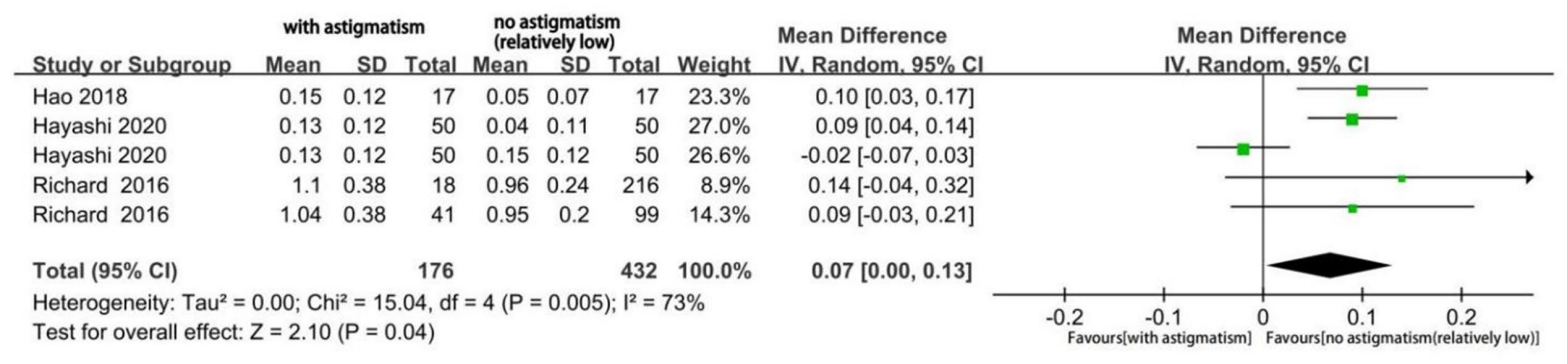
Forest plot of uncorrected intermediate visual acuity (UIVA).

**Figure 9:**
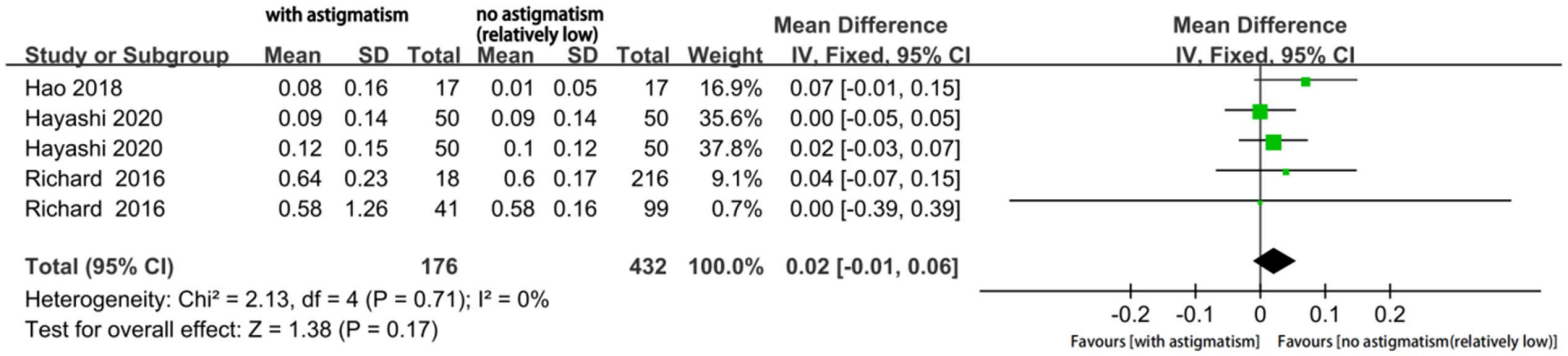
Forest plot of uncorrected near visual acuity (UNVA).

The mean UIVA significantly differed according to the magnitude of astigmatism (MD 0.07; 95% CI, 0.00 to 0.13; *p* = 0.04; Figure 8). The I^2^ value was 73%; moreover, subgroup analysis was performed but did not eliminate the significant heterogeneity. In this case, our sensitivity analysis still showed good stability. Finally, the mean UNVA did not significantly differ according to the astigmatism magnitude (MD: 0.02; 95%CI −0.01, 0.05; *p* = 0.17; Figure 9) and the I^2^ was very low.

## 4 Discussion

Modern cataract surgery with MIOL implantation can allow good spectacle independence and stable vision (17). However, astigmatism, which is among the most common refractive errors in adults worldwide (18), is among the major causes of dissatisfaction following MIOL implantation (19–21). The high prevalence of astigmatism among patients undergoing cataract surgery presents a significant public health challenge (4). Therefore, it is important to elucidate the independent role of astigmatism in the outcomes of MIOL implantation.

Residual astigmatism has been shown to affect VA following MIOL implantation unlike monofocal IOL (22, 23). Additionally, VA has been shown to be negatively correlated with the magnitude of astigmatism, with the decline being more evident in distance VA (14, 16, 24–28). Moreover, a high incidence of astigmatism has been reported in patients with blurred vision following MIOL implantation (19–21). Recent studies have shown that vision is greatly influenced when the magnitude of astigmatism is ≥1.0D and ≥0.75D in eyes with bifocal and trifocal IOLs, respectively (14, 16, 24, 25); however, there remains controversy (27–30).

In our study, most of the included studies did not report the mean VA; therefore, we analyzed the proportion of eyes with a useful postoperative VA according to the magnitude of astigmatism (1.5D, 1.0D, and 0.5D), which revealed significant differences. Previous studies (29) have reported impaired visual quality (optical parameters, patient satisfaction, etc.) even with relatively low astigmatism (<1.0D). Lee et al. (31) found no significant correlation between the aforementioned parameters and VA, which indicates a discrepancy between optical quality and VA in eyes implanted with MIOL. However, we observed a significant difference in VA when astigmatism was 0.5D, indicating a postoperative effect of astigmatism on VA even at low astigmatism levels. Compared with astigmatism with a magnitude of 1.0D and 0.5D, a much more significant difference was observed at a magnitude of 1.5D. Similar results was observed in the analysis of astigmatism with a magnitude of 1.0D and 0.5D, which is consistent with the aforementioned previous reports.

To further confirm this effect, we analyzed residual astigmatism in patients with blurred vision following MIOL implantation. Our findings indicated a correlation between astigmatism and blurred vision in MIOL-implanted eyes, which may contribute towards dissatisfaction following MIOL implantation (19–21). Given the small number of included studies, our analysis was based on astigmatism with a magnitude of >0.75D; however, a significant difference was observed even at a magnitude of <1.0D.

Moreover, we sought to identify the effects of postoperative astigmatism on VA at all distances. Only few studies reported the mean postoperative VA; moreover, most of the studies reported that the MIOL was significantly affected when the magnitude of astigmatism was ≥1.0D. For consistency purposes, we selected studies that considered an astigmatism magnitude of ≈1.0D for comparison with no or relatively low astigmatism. We found that the UDVA and UIVA, but not UNVA, significantly differed according to the astigmatism magnitude (14, 16, 24–28). Although the significant difference was less evident in UIVA than in UDVA, most studies only reported the effect of postoperative astigmatism on UDVA; nonetheless, UIVA is as important in our daily lives as other VAs. The observed discrepancies in VA at different distances could be attributed to the complex multifocal structure and narrow VA peak curve at a long distance (32, 33). This phenomenon is more significant with implantation of trifocal IOLs given the more complex light diffraction in the eyes (14).

This study has several strengths. First, we performed a systematic and comprehensive database search without time restrictions to improve statistical power and reduce publication bias. Further, this study demonstrates the scarcity of relevant research given the publication years and small number of the included. Nonetheless, to our knowledge, this is the first meta-analysis to assess the effects of corneal astigmatism, particularly low-level postoperative astigmatism, on MIOLs.

However, this study has several limitations. First, this is a new and developing research topic; accordingly, there were few relevant studies. Moreover, several related studies were excluded since they only reported the correlation results without providing raw data. Second, there was significant heterogeneity among the included studies, which may be partly attributed to differences in other factors that influence the visual outcomes following MIOL implantation, such as study design, population characteristics, follow-up time, models of MIOL, and outcome measurement. For these multiple factors, it is difficult to detect the source of heterogeneity. However, Figure 3 shows the heterogeneity caused by a study performed in 2000 (16), which is justified because the refractive model of MIOL used in 2000 is not commonly used nowadays.

## 5 Conclusion

Astigmatism is prevalent among patients with cataract and significantly influences UDVA and UIVA following MIOL implantation. Additionally, visual outcomes appeared to be affected when the postoperative astigmatism was ≥0.5D. Therefore, Accurate preoperative and postoperative evaluation of astigmatism is important. Moreover, it should be treated tactfully, including through a corneal relaxing incision or toric, rather than non-toric, IOL implantation to control postoperative astigmatism of ≤0.5D. Further research is warranted to inform guidelines for astigmatism correction in patients undergoing MIOL implantation.

## Data availability statement

The original contributions presented in the study are included in the article/supplementary material, further inquiries can be directed to the corresponding author.

## Author contributions

WS was responsible for conception and design, extracted the data, and wrote the manuscript. WS, BZ, LZ, JS, and DM collected the literature. WS and BZ analyzed the data. JY critically revised the manuscript. All authors contributed to the article and approved the submitted version.
